# Optimization of the Heterogeneous Synthesis Conditions for Cellulose Tosylation and Synthesis of a Propargylamine Cellulosic Derivative

**DOI:** 10.3390/polym17010058

**Published:** 2024-12-29

**Authors:** Marcos V. Ferreira, Poliana Ricci, Henrique A. Sobreira, Anizio M. Faria, Rodrigo B. Panatieri, Brent S. Sumerlin, Rosana M. N. Assunção

**Affiliations:** 1 Institute of Chemistry, Federal University of Uberlândia, Uberlândia 38400-902, Brazil; 2 Institute of Exact and Natural Sciences, Federal University of Uberlândia, Ituiutaba 38304-402, Brazil; 3 George & Josephine Butler Polymer Research Laboratory, Department of Chemistry, University of Florida, Gainesville, FL 32608, USA

**Keywords:** cellulose tosylation, Doehlert matrix statistical design, infrared spectroscopy, alkyne functionalization, aminopropargyl cellulose

## Abstract

Cellulose tosylate (MCC-Tos) is a key derivative for surface modification and a crucial precursor for cellulose compatibilization in click reactions, enabling its functionalization for advanced applications. Replacing tosyl groups with alkyne groups broadens cellulose’s potential in biocompatible reactions, such as thiol-yne click chemistry and protein/enzyme immobilization. To achieve this, we optimized the heterogeneous synthesis of MCC-Tos using a Doehlert matrix statistical design, evaluating the influence and interaction of the reaction conditions. The optimized conditions—144 h reaction time, 10:1 molar ratio, and 30 °C—yielded a degree of substitution for tosyl groups (DS_tos_) of 1.80, determined via elemental analysis and FTIR-ATR spectroscopy. The reaction kinetics followed a first-order model. A subsequent reaction with propargylamine produced aminopropargyl cellulose (MCC-P_NH_), reducing DS_tos_ by 65%, which was confirmed via FTIR, and improving thermal stability by a margin of 30 °C (TGA/DTG). ^13^C CP/MAS NMR confirmed the alkyne group attachment, further validated via coupling an azide-functionalized coumarin through copper(I)-catalyzed alkyne-azide cycloaddition (CuAAC). Fluorescence microscopy and UV spectroscopy were used to estimate a substitution degree of 0.21. This study establishes a feasible route for synthesizing alkyne-functionalized cellulose, paving the way for eco-friendly materials, including protein/enzyme bioconjugates, composites, and advanced materials via thiol-yne and CuAAC reactions.

## 1. Introduction

Due to its unique properties—such as low extraction cost, renewable sourcing, biocompatibility, biodegradability, high elastic modulus, and specific strength—cellulose is widely used in various applications, including gas barrier films, antimicrobial coatings, transparent films, flexible displays, biomedical implants, drug delivery systems, hemodialysis, and electroactive polymers [1,2,3,4,5,6,7,8,9]. These applications enable the development of high-value products using agro-industrial raw materials or their residues as sources for cellulose extraction, which are produced on a large scale globally (an estimated 911 million tons of agricultural residues in 2023) [10]. By utilizing such residues for cellulose production, cellulose demonstrates a significant potential to provide sustainable and renewable alternatives to petroleum-based materials, thereby reducing the dependence on fossil-based sources [11,12].

Cellulose has a linear structure composed of glucose units linked by β-1,4 bonds, resulting in a highly functional and hydrophilic surface due to the presence of hydroxyl groups [13]. However, this linear structure, characterized by inter- and intra-chain hydrogen bonds and its semicrystalline nature, makes cellulose insoluble in water and many organic solvents, posing challenges for its functionalization. The chemical functionalization of cellulose offers a promising way to expand its applications. Modifying cellulose’s hydroxyl groups can alter its mechanical properties, strength, flexibility, and hydrophobicity [4].

Two main routes can be used to synthesize cellulose derivatives: the homogeneous approach, where cellulose is dissolved along with the reagents required for functionalization, and the heterogeneous approach, where cellulose remains dispersed in a liquid phase containing the reagents [2]. The heterogeneous strategy has advantages in terms of retaining some of the native polymer structure and mechanical properties. Thus, it is the preferred route in this work despite typically requiring longer reaction times, high temperatures, and excess reagents [14]. Understanding the reaction kinetics and studying the optimization conditions are paramount to improving the reaction rates and achieving a higher degree of substitution (DS).

Each anhydroglucose unit (AGU) in cellulose contains three hydroxyl groups available for substitution, located at the glucose monomers at carbon 6 (C6), carbon 2 (C2), and carbon (C3) positions, in the order of their reactivity. As a result, higher DS values are achieved when substitution approaches the theoretical maximum of 3 [15].

Due to the differences in reactivities among cellulose’s hydroxyl groups and the inherent limitations of cellulose modifications to reactions based on its “O-chemistry”—i.e., the attack of the oxygen atom donor from the hydroxyl group (OH) on electrophiles—the use of cellulose intermediates, such as cellulose halides, cellulose carbonates, epoxy-grafted celluloses, and cellulose sulfonates, enables the formation of a wide range of substituted products [16,17,18,19]. These intermediates can later react with the nucleophilic groups of interest, where the substituent group will act as a leaving group. The choice of a cellulose intermediate primarily depends on the reaction conditions required for its synthesis and the reactivity of the substituent group [17].

In this work, we focused on cellulose tosylate, a derivative from the class of cellulose sulfonates, due to its particular advantage as a precursor for synthesizing other cellulose derivatives and expanding the material application. Cellulose tosylate features a tosyl group that facilitates subsequent nucleophilic substitutions to introduce a plethora of functional groups into the final material [15,20,21,22,23,24].

Cellulose tosylate is particularly significant in cellulose chemistry due to its ability to undergo nucleophilic substitution (S_N_) reactions, enabling the replacement of tosyl groups with various nucleophilic groups, such as halides, azides, or amines, to produce the respective deoxycellulose derivative [20,23,24,25]. The kinetics of cellulose tosylation are directly related to the DS, which plays a critical role in the efficient functionalization of hydroxyl groups across both the amorphous and crystalline regions of cellulose [14]. Studies have demonstrated that tosylation follows pseudo-first-order kinetics, where the primary hydroxyl groups exhibit a higher reactivity than the secondary ones due to the reduced steric hindrance and greater accessibility [26]. The DS in these reactions is controlled, in part, by the molar ratio (MR) of the derivatizing agent to the anhydroglucose unit (AGU) [27,28]. When the DS exceeds one, nucleophiles exhibit selectivity for hydroxyl groups at the C6 position. This selectivity arises from the steric hindrance at the C2 and C3 positions and is further influenced by the nucleophilicity of the reagent used [20,23].

Despite these insights, the literature on the detailed kinetics of cellulose tosylation remains limited, highlighting the need for further investigation into optimizing the production of cellulose tosylate with the desired DS for specific applications. This study addresses this gap by employing a Doehlert matrix statistical design to optimize the synthesis of cellulose tosylate (MCC-Tos) and analyze its kinetic profile.

To further expand the applications of cellulose in polymer science, we also explored the use of the aminopropargyl group as a nucleophile to replace the tosyl group in MCC-Tos, forming aminopropargyl cellulose (MCC-P_NH_). The introduction of a propargyl group broadens cellulose’s applicability by enabling compatibility with versatile click reactions, such as copper-catalyzed azide-alkyne cycloaddition (CuAAC) and thiol-yne reactions, advancing sustainable chemistry [29,30,31].

Additionally, we synthesized an azide dye to confirm the successful formation of MCC-P_NH_ and evaluated the potential of this cellulosic derivative as a molecular attachment platform via a CuAAC click reaction. This approach was further employed to assess the aminopropargyl groups’ DS and their distribution throughout the cellulose fibers. Through these efforts, this study aimed to optimize the synthesis and functionalization of cellulose derivatives, expanding their applicability in advanced materials and sustainable polymer science.

## 2. Materials and Methods

MCC (Synth^®^, Diadema, Brazil), with a degree of polymerization (DP) of 350, was pretreated by taking the following steps: drying in an oven at 110 °C for 1 h; washing with ethanol (Dinâmica^®^, Indaiatuba, Brazil) and centrifuging (2500 rpm, 3 min each cycle) 4 times; dispersing in 5 mL of *N-N*-dimethylacetamide (DMAc) (Acros-Organics^®^, Geel, Belgium); drying in an oven at 110 °C for 20 h; and storing in a desiccator until further use [32]. Pyridine (Synth^®^, Diadema, Brazil) and dimethylsulfoxide (DMSO) (Synth^®^, Diadema, Brazil) were purified according to the procedures of Yoshikawa et al. (2010) [33,34]. The water used in all the experiments was high-purity water (ASTM type I, with resistivity ≥18.3 MΩ cm) obtained from a Megapurity^®^ ultrapurification system (Billerica, MA, USA). Acetone was purchased from Synth^®^ (Diadema, Brazil) and used as received. Propargylamine and *p*-toluenesulfonyl chloride were purchased from Sigma-Aldrich^®^ (Cajamar, Brazil) and used as received.

### 2.1. General Procedure for Cellulose Tosylation

In a vial with a septum screw cap, 0.20 g (1.23 mmol) of MCC was dispersed in 10 mL of pyridine and stirred magnetically overnight after a 5 min purge under an argon flow (White Martins^®^, Rio de Janeiro, Brazil) [35]. The appropriate weight of *p*-toluenesulfonyl chloride (TosCl) was dissolved in 1 mL of pyridine and added dropwise through the septum using a hypodermic syringe with a 25 × 7 mm needle [36]. The variations in the reaction time, molar ratio, and temperature were investigated. The obtained product was centrifuged to remove excess reagents and redispersed in a 1:1 mixture of ultrapure water and acetone, followed by vortexing for 30 sec. This centrifugation was repeated thrice at 2500 rpm for 3 min each. The resulting material was washed four times with ultrapure water, dried in an oven at 60 °C for 20 h, and stored in a desiccator until further use.

### 2.2. Optimization of the Tosylation Reaction via Statistical Design

The selection of a Doehlert matrix for the experimental design comes from its capability to prioritize the factors deemed the most relevant in a study by assigning them more levels. This approach allows for the simultaneous evaluation of the interactions between factors without requiring an exhaustive number of experiments, thereby reducing the costs, time, and waste generation [37]. In the studies on the heterogeneous tosylation of cellulose, Heuser et al. (1950) identified evidence that the reaction time and molar ratio had the most significant impact on the degree of substitution [26]. Therefore, in this work, we chose to investigate the optimization of both the molar ratio and reaction time in the tosylation synthesis while also including temperature, as it appeared to influence another tosylation process [23]. Since the reaction time (x_2_) factor seemed to have more impact on the DS_tos_, we considered it the most critical and assessed it at the highest number of levels (seven). To enable a thorough examination of the temperature (x_1_) influence, we evaluated this factor at five distinct levels, ranging from 30 to 90 °C. Finally, the molar ratio (x_3_) was assessed at three levels. Optimization experiments were carried out by combining the factors and levels described in Table 1.

The levels in Table 1 represent the actual values of the factors evaluated in optimizing the tosylation reaction. These exact values were coded according to Equations (1)–(3) for statistical analysis.

(1) $$x_{1}=\frac{(T\;-60)}{30}$$

(2) $$x_{2}=\frac{(t-38)}{41.570}$$

(3) $$x_{3}=\frac{(\mathrm{MR}-5)}{2.449}$$

To follow the tosylation progress, we adapted a method devised by Rahn et al. (1996) based on the correlation of the absorbance ratio (AR) for absorptions at 1174 cm^−1^ (attributed to the SO_2_ bond from the tosyl group) and at 1056 cm^−1^ (corresponding to C-O-C stretching in the cellulose backbone) [36]. These measurements were obtained via FTIR-ATR analysis and later were correlated with the sulfur percentage determined by elemental analysis to obtain the respective degree of substitution (DS_tos_) (see elemental analysis section, Table S1 and Figure S1 of the Supplementary Materials). This approach allowed us to use a fast, reliable, and cost-effective technique, such as FTIR-ATR spectroscopy, to estimate the AR and DS_tos_ for the tosylated samples.

### 2.3. Aminopropargylation of the Tosyl-Cellulose Samples

We dissolved 0.72 g of tosyl-cellulose (1.64 mmol, DS_tos_ 1.80) in 36 mL of DMSO (Synth®, Diadema, Brazil) and carefully added 2.66 g (50.2 mmol, 30:1) of propargylamine (Sigma-Aldrich, Cajamar, Brazil). The mixture was stirred for 168 h at 60 °C and then cooled to room temperature. The suspension was precipitated into 100 mL of acetone and washed thrice with 50 mL of cold water. Aliquots were taken periodically to monitor the reaction’s progress. After washing, the products were dried under a vacuum at 45 °C [29].

### 2.4. Characterization

FTIR-ATR absorbance spectra were recorded using an Agilent Cary 630 FTIR spectrometer (Wilmington, NC, USA), with acquisitions spanning the 4000 to 650 cm^−1^ range. The spectra were obtained at a resolution of 4 cm^−1^, involving 128 scans for each sample and 128 background scans. Solid-state NMR ^13^C CP/MAS spectra were acquired on a Bruker Avance III HD (Ettlingen, Germany) nuclear magnetic resonance spectrometer, operating in a magnetic field of 7.05 T, equipped with a double resonance probe and a zirconia MAS rotor with a 4 mm outer diameter. UV–vis spectra were obtained using the Molecular Devices SpectraMax M2 (San Jose, CA, USA) multimode instrument in the 200–800 nm range, using a quartz cell with a 1.0 cm optical path.

TGA/DTG curves were collected using a TA Instruments TGA55 Thermal Analyzer (New Castle, DE, USA), with all the experiments conducted under a nitrogen flow of 60 cm^3^ min^−1^. Approximately 5 mg of each sample was placed in an open 100 µL platinum sample holder and heated at 10.0 °C min^−1^ over a 30–600 °C temperature range. Elemental analyses (EA) were performed using a Thermo Scientific Flash EA 1112 (Milan, Italy) elemental analyzer.

Fluorescence microscopy images were captured using a Nikon ECLIPSE Ti2 fluorescence inverted microscopy system (Kanagawa, Japan) featuring a 25 mm field of view (FOV) and EmuNikonTi22911#(Kanagawa, Japan).

## 3. Results and Discussion

### 3.1. Optimization of the Tosylation Reaction

**Scheme 1:**
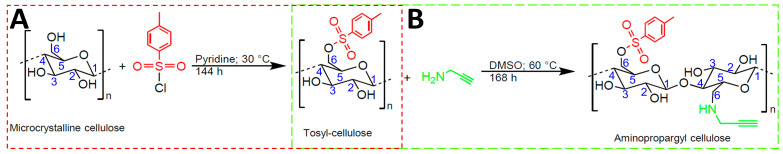
(**A**) Synthetic approach toward the obtention of tosyl-cellulose (MCC-Tos); (**B**) synthetic route for the obtention of aminopropargyl-cellulose (MCC-PNH) from MCC-Tos.

To explore the reactivity of the cellulose hydroxyl groups and synthesize an alkyne derivative able to participate in thiol-yne click reactions, the strategy that we adopted was to first synthesize a derivative intermediate, tosyl-cellulose (MCC-Tos), through the reaction presented in Scheme 1A.

Although the tosylation reaction of cellulose under heterogeneous conditions (Scheme 1A) is well known and established, optimizing these conditions remains an essential area of study for understanding cellulose chemical surface modifications—the few examples of cellulose tosylation optimization in the literature focus on the homogeneous phase. For instance, Hamdaoui et al. (2020) demonstrated that a statistical design of experiments can be a valuable tool for optimizing cellulose modification reactions [24]. In their work, the authors used a Box–Behnken Design (BBD) to assess the effect of three factors (TosCl equiv, amount of base, and reaction time), in which they identified the number of tosyl chloride mols as the most influencing factor.

Here, we adopted a Doehlert matrix of statistical design due to its ability to simultaneously evaluate various factors that may affect the tosylation reaction of cellulose while still enabling the prioritization of the most critical variables in the study. During the investigation of cellulose tosylation, the reaction time (x_2_) proved to be the most significant factor. Therefore, it was assessed at seven different levels to evaluate its impact. The temperature factor (x_1_) was investigated at five different levels to thoroughly examine the 30 to 90 °C range, while the molar ratio factor (x_3_) was evaluated at three levels. Then, the three factors (molar ratio, temperature, and reaction time) were coded and combined in fifteen experiments, as displayed in Table 2, along with the response factor AR obtained from the FTIR spectra of the tosylated samples (see Figure S2 in the Supplementary Materials).

The experimental conditions described in Table 2 resulted in AR responses ranging from 0.302 to 0.535, with a standard deviation of 0.0269 and a coefficient of variation of 6.20% for the triplicate measurements at the center point. These values indicate that the synthesis method and the FTIR spectroscopy measurements exhibited good repeatability. Therefore, the software Design-Expert^®^ (trial version #13, Stat-Ease, Inc., Minneapolis, MN, USA) was used to statistically analyze the data from Table 2 and establish the relationship between the molar ratio, time, and temperature by defining a mathematical model to predict the variable conditions that yield the highest efficiency of the MCC tosylation reaction. Table S2 (see the Supplementary Materials) presents the values of the coefficients obtained from the mathematical model.

A coefficient was considered statistically significant if the *p*-value was less than 0.05 at a 5% significance level. Thus, we wrote Equation (4) based on these significant parameters to describe the optimization of the tosylation reaction.

AR (a.u.) = 0.437 + 0.088x_2_ + 0.075x_3_ (4)

According to Equation (4), the factors of time (x_2_) and molar ratio (x_3_) were significant, which justified the need for experimental planning to understand their interaction. The statistical validity of this model was confirmed by the Analysis of Variance (ANOVA), presented in Table 3.

According to the ANOVA in Table 3, the quadratic model of Equation (4) fits well with the experimental results of the MCC modification through the heterogeneous phase tosylation reaction. This fit is confirmed by the statistical significance (*p* < 0.05) at a 95% probability level, as determined by the F-test of the regression model. Assuming a significance level of α = 0.05, the critical F-value for df1 = 9 and df2 = 5 is approximately 4.77. Since the calculated F-value (9.70489) exceeds the critical value, it indicates that the regression model is statistically significant. Although the model shows a lack of fit, it explains 94.6% of the variations observed in the experiment (R^2^_adjusted_) and accounts for 99.93% of the maximum explainable variations (R^2^). These percentages were obtained using Excel^®^ spreadsheets for data processing from the Doehlert matrix provided by Teófilo and Ferreira (2006) [37].

Figure S3 shows a plot of the predicted AR against the experimental results. The regression line demonstrates that the experimental and expected results are very close (R^2^ = 0.9417). Consequently, it can be concluded that the data fall within the confidence limits and that the model adequately fits the actual data.

### 3.2. Estimated Response Surface and Contour Plots

**Figure 1:**
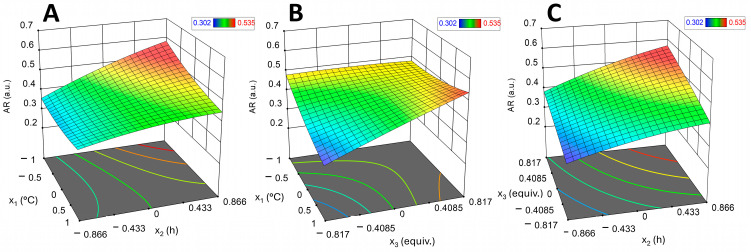
Three-dimensional response surfaces and contour plots for the Doehlert matrix at encoded values of (0,0,0): (**A**) molar ratio (x_3_) = 5 equiv (TosCl); (**B**) time (x_2_) = 38 h; (**C**) temperature (x_1_) = 60 °C.

The proposed quadratic model and the interactions between the variables, whether significant or not, can be better interpreted through the three-dimensional response surface plots shown in Figure 1.

Figure 1A shows that at an intermediate molar ratio of 5 equiv, lower temperatures and longer reaction times significantly increase the efficiency of tosylation reactions, achieving the highest predicted AR value of 0.582 under these conditions. However, as seen in Figure 1B, higher temperatures and molar ratios were required when the reaction time was limited to a maximum of 38 h. Yet, the highest predicted AR value did not exceed 0.539. In Figure 1C, when the temperature was maintained at 60 °C, higher AR values, such as 0.570, were obtained only at longer reaction times and high molar ratios. Therefore, the statistical model indicated that under the conditions of high molar ratio and long reaction time, temperature does not significantly influence the adduct formation, highlighting the statistical significance of the x_2_ and x_3_ parameters in the heterogeneous tosylation of cellulose.

In summary, the higher efficiency of tosylation at longer reaction times (74 h) and increased amounts of reagents (7:1—TosCl:AGU) may be due to the difficulty in accessing and making available the hydroxyl groups of cellulose in a heterogeneous medium. This conclusion is further supported by the observation that tosylation reactions preferentially substitute at the C6 position under these conditions, even without pronounced regioselectivity for low and medium DS_tos_ values. These findings significantly affect the designing and optimizing of tosylation reactions in practical applications [38,39]. Concerning the temperature factor, higher temperatures seemed insignificant for this type of reaction, as they did not ensure a considerable increase in the diffusion of reagents and their interaction with the most reactive regions of the AGU. Thus, near-ambient temperatures were chosen as the reaction conditions for tosylation in this work.

The optimized conditions were a reaction time of 74 h, a molar ratio of 7:1 (TosCl:AGU), and a temperature of 30 °C. The sample produced under these conditions was named MCC-Tos 7:1 (74 h) and presented an AR value of 0.647, confirming the reasonable predictability of the experimental design, as the theoretically calculated value for these optimized conditions was 0.595 ± 0.027. Considering the method for DS_tos_ determination described in Section S1 of the Supplementary Materials, we estimated a DS_tos_ equal to 1.04 for this sample.

Applying an experimental design, such as the Doehlert matrix, allowed the optimized conditions to be extrapolated to values beyond the evaluated intervals [37]. Thus, the reaction time was extended to 144 h, the molar ratio was increased to 10:1 (TosCl:AGU), and the temperature was kept constant at 30 °C. This sample, MCC-Tos 10:1 (144 h), presented an almost twofold increase in the AR value, reaching 1.016 and a DS_tos_ of 1.80 (see Figure S4 in the Supplementary Materials).

Table 4 presents a comparative analysis of the DS_tos_ achieved in cellulose tosylation reactions using various methodologies, reaction media, and conditions.

The DS_tos_ values vary significantly depending on the reaction route (heterogeneous or homogeneous), the reaction medium, the molar ratio of tosyl chloride to the anhydroglucose unit (TosCl:AGU), temperature, and reaction time.

Heterogeneous reactions using pyridine as the solvent generally achieved a moderate to high DS_tos_. For example, Takahashi et al. (1986) reported a DS_tos_ of 1.40 using a 3:1 molar ratio at 100 °C for 90 h, while Heuser et al. (1950) achieved a slightly higher DS_tos_ of 1.52 under milder conditions (25 °C, 50 h, with a higher molar ratio of 10:1) [26,41]. In contrast, the heterogeneous reaction combining pyridine and an ionic liquid (AMIMCl) at 25 °C for 24 h resulted in a lower DS_tos_ of 0.72 (Gericke et al., 2012), highlighting the influence of the solvent system on the tosylation efficiency [20].

Homogeneous routes, particularly those using solvents like NaOH/H₂O and LiCl/DMAc, generally provide higher DS_tos_ values. Elchinger et al. (2012) achieved a DS_tos_ of 1.70 using NaOH/H₂O with a high TosCl:AGU ratio of 12:1 at 25 °C for 24 h, demonstrating the effectiveness of homogeneous conditions [40]. Heinze et al. (1996) reported the highest DS_tos_ of 2.02 using LiCl/DMAc at low temperatures (5 °C) with a 6:1 molar ratio, underscoring the efficiency of homogeneous reactions in enhancing the substitution levels [22].

The tosylation approach from this study achieved a DS_tos_ of 1.80 under heterogeneous conditions with pyridine at 30 °C over 144 h using a 10:1 molar ratio. This highlights the optimization of the reaction parameters to maximize the substitution while still employing a heterogeneous route, bridging the gap between the moderate substitutions seen with pyridine and the higher substitutions of homogeneous methods.

### 3.3. Kinetic Evaluation for the Tosylation Reaction

To address the indications of a rather pivotal kinetic component in the cellulose heterogeneous tosylation reaction, we investigated the kinetic modeling by experimentally monitoring the progress of the reaction through the DS_tos_ values, using the conversion parameter of the tosylate substitution of the native hydroxyls as a function of time [42]. For this study, the degree of conversion was monitored at different times up to 216 h for a reaction with a 5:1 (TosCl:AGU) molar ratio and a temperature of 30 °C (see Figure S5 of the Supplementary Materials). Also, it was assumed that only a fraction of the primary hydroxyl sites, preferably at C6, would be available for the reaction.

According to the experiment, the maximum degree of substitution (DS_max_) reached upon equilibrium was 1.09, which corresponded to the maximum number of anhydroglucose unit moles consumed (n_AGUmax_). Thus, we used Equation (5) to obtain the total conversion (X_A_) of hydroxyls to tosyl groups [43].

(5) $$X_{A}=\frac{\left(n_{\mathrm{AGUmax}}-n_{\mathrm{AGU}}\right)}{n_{\mathrm{AGUmax}}}$$

where X_A_ is the total conversion, and n_AGU_ is the number of moles of hydroxyl groups substituted by tosyl groups per anhydroglucose unit.

The conversion stabilized after 144 h, indicating that equilibrium had been reached. Three pseudo-order models, zero, first, and second order (Figure S6), were tested to understand the reaction kinetics within this period. Each model’s determination coefficient (R^2^) was calculated, with values closer to 1 indicating a better fit to the experimental data.

The R^2^ value was approximately 0.797 for the pseudo-zero-order model, indicating an inadequate fit. Similarly, the pseudo-second-order model yielded an R^2^ of 0.680, suggesting a poor fit. These results demonstrate that neither the pseudo-zero-order nor pseudo-second-order models adequately represented the experimental data.

Conversely, the pseudo-first-order model provided an R^2^ value of 0.945, indicating an excellent fit to the experimental data. This result suggests that within the first 144 h of the reaction, the kinetics follow a first-order model, where the reaction rate is directly proportional to the concentration of the reactant, further proving the significance of the molar ratio factor on the DS_max_. Therefore, the reactant concentration significantly influences the reaction rate during this period. This behavior is typical in the kinetics of cellulose substitution reactions [14,44].

The pseudo-first-order rate law for the cellulose tosylation in the heterogeneous phase can be expressed as:

(6) $$\mathrm{rate}=K_{b}\;\left[\mathrm{tosyl}\;\mathrm{cellulose}\right]=K_{a}\;\left[\mathrm{cellulose}\right]\left[\mathrm{tosyl}\right]$$

and with the concentration of cellulose in excess, it remains effectively constant.

The model’s rate constant (K_b_) was determined to be 0.01774 h^−1^, indicating slow reaction kinetics. This highlights the significance of the time factor and the ineffectiveness of increasing the temperature [42]. This phenomenon can be partly attributed to the MCC’s crystallinity, which hinders the exposure of the hydroxyl groups from the crystalline region. These regions slow down the reactant diffusion while the reaction progresses in the amorphous phase [14]. Additionally, chemical surface modifications in the heterogeneous phases are known to occur erosively, starting from the top and working down through the crystalline regions, and thus are further influenced by the hydroxyl availability [14,44].

The tosylation reaction of cellulose is not strictly regioselective, but it has higher esterification rates at the C6 hydroxyl groups compared to those at C2 and C3. Under heterogeneous reaction conditions, the relative reaction rates are such (5.8 times higher for C6 than for C2) that it is impossible to replace primary hydroxyl groups with tosyl groups without substituting a certain number of secondary hydroxyl groups [26]. The different reactivities among the hydroxyl groups present in the cellulose structure must be considered, which leads to the preferential formation of a derivative with a certain degree of substitution [4,15,22,39].

Furthermore, as we used pyridine as both a swelling and base reagent to facilitate the accessibility of the hydroxyl, selected a high molar ratio (10:1, TosCl:AGU), and provided extended reaction times up to 144 h, our optimized tosyl-cellulose MCC-Tos 10:1 (144 h) reached DS_tos_ values (1.80) higher than the previous informed in the literature of 1.52 for heterogeneous reactions (Table 4) [26].

### 3.4. Aminopropargylation of the Tosyl-Cellulose

The next step in our strategy to produce a cellulose derivative compatible with a thiol-yne click reaction involved using the propargylamine as a nucleophile to replace the tosyl groups in the optimized MCC-Tos. This approach enables the insertion of an alkyne group by substituting the tosyl groups, which act as leaving groups in this reaction. This method yields a cellulose derivative that cannot be synthesized directly by reacting with the cellulose hydroxyls. To achieve that, MCC-Tos (DS_tos_ = 1.80) was reacted with 30 equiv of propargylamine over 7 d under constant stirring at 60 °C (Scheme 1B).

**Figure 2:**
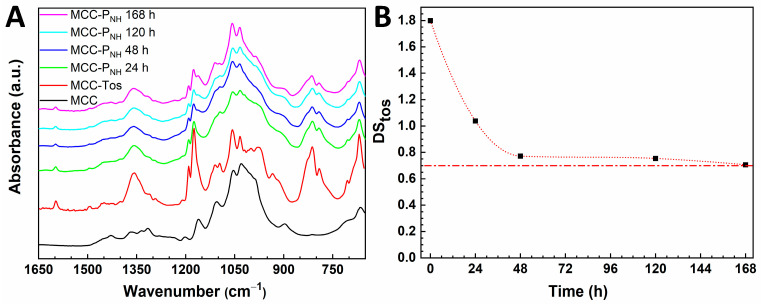
(**A**) MCC, MCC-Tos, and MCC-P_NH_ 24 to 168 h FTIR spectra in the 1650 to 650 cm^−1^ range. (**B**) Plot of DS_tos_ as a function of propargyl reaction time.

Aliquots of the reaction were periodically taken to monitor the progress by observing the changes in the intensities of the FTIR bands related to the tosyl groups. Significant changes in the aminopropargyl cellulose (MCC-P_NH_) spectra, particularly the decrease in the intensities of the bands associated with the tosyl groups (ν_assim_SO_2_ in 1360 cm^−1^; ν_sim_SO_2_ in 1174 cm^−1^; ν_aromatic ring_ in 834 cm^−1^), are evident in Figure 2A.

However, no clear evidence of bands associated with the amino or propargyl groups was present. Therefore, we used the linear correlation expressed in Equation (S1) to calculate the DS_tos_ to obtain a more quantitative approach to the number of tosyl groups removed. The results are shown in Figure 1B and point to a decrease of around 61% in the initial number of tosyl groups. No significant changes occurred after 48 h, indicating that the reaction reached a steady plateau after 168 h of reaction.

### 3.5. Thermogravimetric Analysis

**Figure 3:**
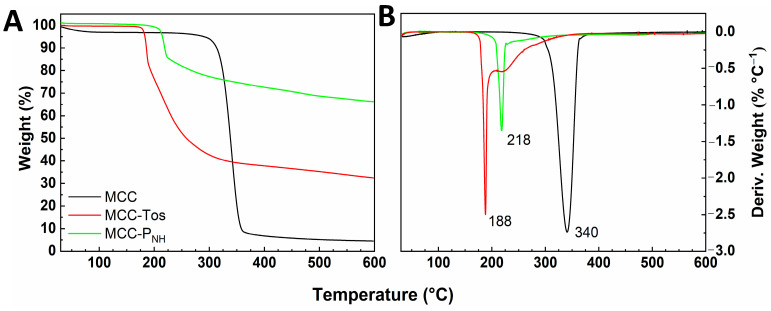
(**A**) TGA curves; and (**B**) DTG curves for MCC, MCC-Tos, and MCC-P_NH_ 168 h.

The FTIR results did not confirm the presence of aminopropargyl grafted onto the MCC-P_NH_. However, they indicated a reduction in the tosyl groups attached to the polymer. To further characterize the final product, we performed a comparative analysis using TGA/DTG analysis, with the results presented in Figure 3.

From Figure 3, the TGA/DTG curves exhibit a slight increase in the thermal stability of MCC-P_NH_ compared to MCC-Tos during the first thermal event, as indicated by the rise in the T_onset_ from 181 to 211 °C and an increase of approximately 30 °C in the T_max_. This event is related to the cleaving of the tosyl groups grafted onto the cellulose main chain, so the increase in thermal stability suggests that fewer tosyl groups are present on MCC-P_NH_, confirming the FTIR results [24].

Additionally, since the degradation products of tosyl groups auto-catalyze the thermal decomposition of the cellulose backbone, the approximately 41% difference in residues indicates that lower amounts of tosyl groups are present in the MCC-P_NH_ sample [22].

### 3.6. CP/MAS ^13^C-NMR Spectroscopy

**Figure 4:**
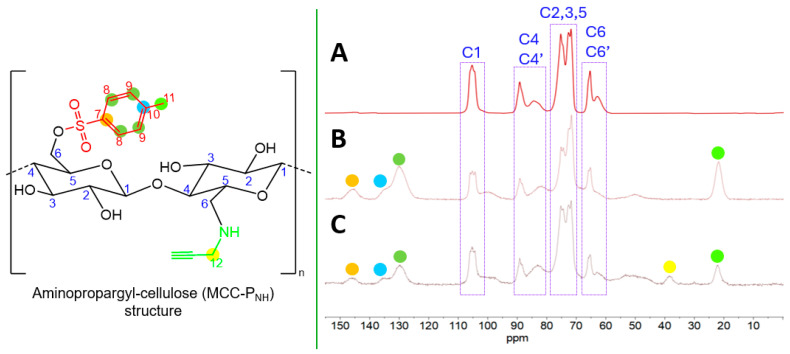
CP/MAS ^13^C-NMR spectra of (**A**) cellulose (MCC); (**B**) tosyl-cellulose (MCC-Tos); and (**C**) amino propargyl-cellulose (MCC-P_NH_) after 168 h of amino propargylation reaction.

The samples were characterized by solid-state ^13^C-NMR spectroscopy to gain more structural information from the synthesized cellulose derivatives. Figure 4 presents the spectra of the original cellulose sample and the cellulose derivates synthesized in this work.

The MCC spectrum exhibited one peak at δ 104.5 ppm, corresponding to the structure’s anomeric carbon (C1). For carbons 2, 3, and 5, three peaks appeared between δ 74.2 and 70.8 ppm [45]. The signals at δ 88.1 and 83.4 ppm are relative to C4, while those at δ 64.3 and 61.6 ppm are characteristic of C6. The appearance of two signals related to C4 (C4 and C4′) and C6 (C6 and C6′) carbons indicates a mixture of crystalline and amorphous cellulose phases in the sample. The peak at δ 88.1 ppm (C4) refers to the crystalline region, while the broader signal at δ 83.4 ppm (C4′) represents the amorphous region. Similarly, the crystalline and amorphous signals C6 and C6′ appear at δ 64.3 and 61.6 ppm, respectively [46].

In addition to the characteristic signals of cellulose, the MCC-Tos spectrum showed additional peaks at around 145, 129, and 20 ppm, corresponding to aromatic carbons associated with the presence of the tosylate group. The signal at 145 ppm can be attributed to the carbon bonded directly to the sulfur atom, while the shoulder observed at around 135 ppm corresponds to the carbon bonded to the methyl group. The peak at 129 ppm represents the other carbon atoms of the aromatic ring, and the methyl group linked to the aromatic ring is indicated at 20 ppm [23,46]. This result confirms the successful grafting of the tosyl group onto cellulose.

In the MCC-P_NH_ spectrum, the appearance of a signal at δ 40 ppm, attributed to the propargyl carbon, provided the first direct evidence of the formation of a propargyl-cellulose derivative. However, the signals attributed to the tosyl groups indicate that the propargylation reaction did not completely replace all the tosyl groups with aminopropargyl groups. The FTIR spectra and TGA/DTG curves also demonstrated the presence of the tosyl groups.

### 3.7. UV–vis and Fluorescence Microscopy Analysis

**Scheme 2:**
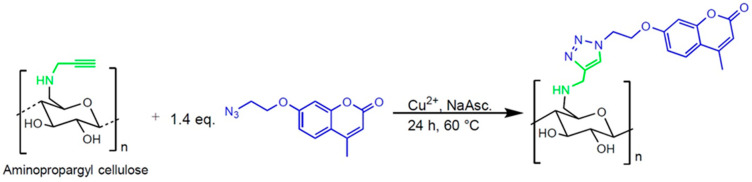
Synthetic route to obtention of the azide-coumarin attached to MCC-P_NH_ via CuAAC reaction.

To further confirm the presence of alkyne groups grafted onto the polymer and quantify the degree of substitution of aminopropargyl groups (DS_NH_), we synthesized an azide-coumarin and used it as a chromophore, attaching it to the MCC-P_NH_ through a copper(I)-catalyzed alkyne-azide cycloaddition (CuAAC) click reaction (Scheme 2). This product was then characterized using UV–vis and Fluorescence Spectroscopy [47,48].

We employed a site-specific CuAAC click reaction to ensure that the azide dye could only be attached through an alkyne group—absent in both native cellulose and MCC-Tos—thereby providing robust evidence for aminopropargyl groups.

The MCC-P_NH_/azide-coumarin product was solubilized in dimethyl sulfoxide (DMSO) and analyzed via UV–vis spectroscopy in the 200 to 500 nm range, with the azide dye’s λ_max_ observed at 315 nm. The UV–vis spectra presented in the Supplementary Materials (Figures S8 and S9) confirmed the successful attachment of azide-coumarin to the polymer, providing definitive proof of propargyl groups on the MCC-P_NH_. The concentration of azide-coumarin grafted onto the polymer, calculated from a standard curve (Figure S8), was 6.90 × 10^−3^ mg mL^−1^. Based on the initial weight of MCC-P_NH_, the degree of substitution for the aminopropargyl group was estimated to be 0.21.

**Figure 5:**
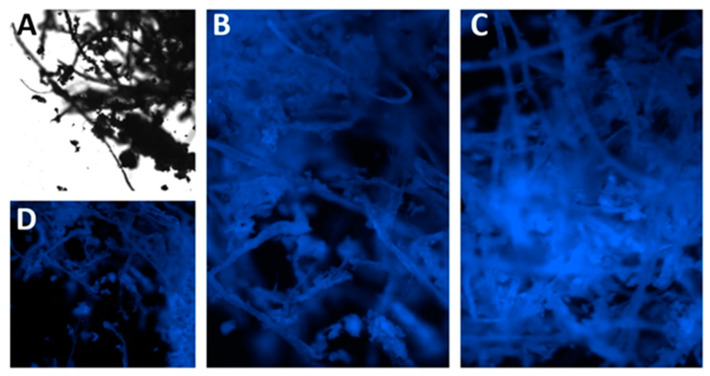
Fluorescence microscopy images of MCC-P_NH_ after azide-coumarin attachment: (**A**) wide-field image; and (**B**–**D**) images for different regions of the same sample, excited at 315 nm with emission at around 430 nm.

Further investigation of MCC-P_NH_ formation was obtained via fluorescence microscopy images, as shown in Figure 5.

In the wide-field image of Figure 5A, the structure of filaments and aggregates characteristic of the unmodified cellulosic material is readily apparent. Figure 5B–D show emissions from the MCC-P_NH_/azide-coumarin product at around 430 nm when excited at 315 nm, which indirectly confirms the presence of alkyne groups. Additionally, the images reveal the uniform distribution of the azide dye throughout the MCC-P_NH_ fibers.

Therefore, optimizing the cellulose tosylation reaction in a heterogeneous phase, followed by the reaction with propargylamine, has proven to be an effective synthetic route for producing MCC-P_NH_. These findings expand the potential for synthesizing novel cellulose-based materials using click reactions such as CuAAC and thiol-yne, which enable the incorporation of a wide range of molecules and macromolecules, requiring only the presence of the corresponding azide or thiol counterparts. Examples of cellulosic-based platforms developed through click reactions include antibacterial cellulose fibers, nanocomposites with barrier properties, drug-delivery vehicles, phosphorescent materials, and the support for immobilizing biomolecules [49,50,51,52,53].

This approach significantly enhances the toolbox for cellulose modification, extending beyond traditional etherification and esterification reactions.

## 4. Conclusions

This study demonstrates that the optimal efficiency of the tosylation reaction, based on the conditions that we investigated, can be achieved under specific conditions: a tosylate to anhydroglucose unit (AGU) molar ratio of 7:1, a reaction time of 74 h, and a temperature of 30 °C. The statistical design suggests that extending the reaction time and increasing the molar ratio could further enhance the degree of substitution (DS). For example, a reaction conducted over 144 h with a 10:1 molar ratio (TosCl:AGU) yielded a DS of 1.80 for MCC-Tos, indicating successful functionalization. The tosyl groups attached to the cellulose acted as effective leaving groups, allowing the subsequent introduction of nucleophilic aminopropargyl moieties onto the cellulose backbone, forming aminopropargyl cellulose (MCC-P_NH_). Successful aminopropargylation was confirmed through various analytical techniques, including FTIR spectroscopy, TGA/DTG, solid-state ^13^C-NMR spectroscopy, and UV–vis spectroscopy. The obtained MCC-P_NH_ displayed reduced thermal stability compared to MCC-Tos, consistent with removing tosyl groups and introducing aminopropargyl groups.

Moreover, the study demonstrated the potential of MCC-P_NH_ to serve as a platform for attaching molecules via CuAAC click reactions. The successful attachment of an azide-coumarin dye confirmed the presence of alkyne groups on the cellulose, with the degree of substitution of aminopropargyl groups estimated to be 0.21. Fluorescence microscopy showed a uniform distribution of the groups through the cellulose fibers.

In summary, this research highlights the feasibility and versatility of chemically functionalizing cellulose, enabling the creation of high-value materials from an abundant and renewable polymer obtained from natural sources. The synthesis of these cellulose derivatives opens new avenues for developing innovative and eco-friendly materials, including protein/enzyme bioconjugates, composites, and other advanced functional materials. 

## Figures and Tables

**Table 1:** Evaluation of factors and levels on the cellulose tosylation reaction.

Factors	Levels						
x_1_ Temperature (°C)	30	45	60	75	90	–	–
x_2_ Time (h)	2	14	26	38	50	62	74
x_3_ Molar ratio TosCl:AGU	3:1	5:1	7:1	–	–	–	–

**Table 2:** Coded values, actual values, and the absorbance ratio for absorptions at 1174/1056 cm^−1^ (AR) according to the Doehlert matrix experiments.

Exp.	Temperature		Time		Molar Ratio		AR
	x_1_	°C	x_2_	h	x_3_	TosCl:AGU	
1 *	0	60	0	38	0	5:1	0.442
2	1	90	0	38	0	5:1	0.434
3	0.5	75	0.866	74	0	5:1	0.488
4	0.5	75	0.2887	50	0.8165	7:1	0.506
5	−1	30	0	38	0	5:1	0.476
6	−0.5	45	−0.866	2	0	5:1	0.328
7	−0.5	45	−0.2887	26	−0.8165	3:1	0.423
8	0.5	75	−0.866	2	0	5:1	0.354
9	0.5	75	−0.2887	26	−0.8165	3:1	0.302
10	0	60	0.5774	62	−0.8165	3:1	0.385
11	−0.5	45	0.866	74	0	5:1	0.535
12	−0.5	45	0.2887	50	0.8165	7:1	0.529
13	0	60	−0.5774	14	0.8165	7:1	0.442
14 *	0	60	0	38	0	5:1	0.437
15 *	0	60	0	38	0	5:1	0.433

* Center point performed in triplicate.

**Table 3:** Analysis of variance (ANOVA) for the Doehlert matrix design.

Source	Sum of Squares	Degrees of Freedom (df)	Mean Square	F-Value	*p*-Value
Regression	0.0633	9	0.007	9.70489	0.01107 *
Residual	0.0036	5	0.0007		
Lack of Fit	0.0036	3	0.0012	50.3405	0.01954 *
Pure Error	5 × 10^−5^	2	2 × 10^−5^		
Total	0.0669	14			
% explained variance			94.5855		
% max. of explainable variance			99.9292		

* Significant at the 95% confidence level (*p* < 0.05).

**Table 4:** Comparison of the degrees of substitution in cellulose tosylation reactions (DS_tos_) synthesized in this work and by using different methodologies.

Reaction Medium	Reaction Route	Molar Ratio (TosCl:AGU)	Temperature (°C)	Reaction Time (h)	DS_tos_	Reference
LiCl/DMAc	Homogeneous *	6:1	5	24	2.02	Heinze et al. (1996) [22]
NaOH/H_2_O	Homogeneous *	12:1	25	24	1.70	Elchinger et al. (2012) [40]
Pyridine	Heterogeneous	10:1	25	144	1.80	This work
Pyridine	Heterogeneous	10:1	25	50	1.52	Heuser et al. (1950) [26]
Pyridine	Heterogeneous	3:1	100	90	1.40	Takahashi et al. (1986) [41]
Pyridine and AMIMCl **	Heterogeneous	3:1	25	24	0.72	Gericke et al. (2012) [20]

* Triethylamine was used as a base in all the homogeneous strategies. ** AMIMCl: 1-allyl-3-methylimidazolium chloride.

## Data Availability

The original contributions presented in this study are included in the article/supplementary material. Further inquiries can be directed to the corresponding author.

## Supplementary Materials

The following supporting information can be downloaded at https://www.mdpi.com/article/10.3390/polym17010058/s1: additional elemental analysis and Doehlert matrix data, along with FTIR, CP/MAS ^13^C-NMR spectra, and UV–vis spectra. It also features graphs showing the estimated degree of substitution (DS) over time for MCC tosylation at a 5:1 molar ratio (TosCl:AGU) and the evaluation of three pseudo-order kinetic models. Table S1. Estimated degree of substitution by elemental analysis of some selected tosyl cellulose samples and their respective absorbance ratio (AR) for the absorptions at 1174/1056 cm^−1^, Figure S1. Absorbance ratio for absorptions at 1174/1056 cm^−1^ as a function of the estimated degree of substitution obtained by elemental analysis of the sulfur content (correlation coefficient 0.9784), Figure S2. FTIR spectra in the 1250 to 950 cm^−1^ range for the tosyl celluloses synthesized under the different experimental conditions established by the Doehlert Matrix. R represents cellulose at 1174 cm^−1^ and Tos or H at 1056 cm^−1^, Table S2. Estimated coefficients of the quadratic model were obtained according to the Doehlert matrix for cellulose tosylation optimization, Figure S3. A plot of the absorbance ratio at 1174/1056 cm-1 as predicted by the Doehlert Matrix versus the values observed in the FTIR spectra (linear correlation of 0.9417), Figure S4. FTIR spectrum for the tosyl cellulose synthesized at room temperature with a reaction time of 144 h and a molar ratio of 10:1 (TosCl:AGU), Figure S5. The behavior of the absorbance ratio at 1174/1056 cm^−1^ and the estimated DS values over time for MCC tosylation at a 5:1 molar ratio (TosCl:AGU), Figure S6. The application of three pseudo-order models: (A) zero, (B) first, and (C) second-order for the tosylation of MCC after reaching equilibrium at 144 h, Figure S7. CP/MAS ^13^C-NMR spectra of amino propargyl-celluloses (MCC-P_NH_): (A) after 24 h; (B) 48; and (C) 168 h of amino propargylation reaction, Figure S8. (A) UV-vis spectra for different concentrations of azide-coumarin; (B) standard curve for the azide-coumarin in mg mL^−1^, with r and R^2^ equal to 0.99949 and 0.99863, respectively, Figure S9. Comparative UV-vis spectra for a 5.4 × 10^−3^ mg mL^−1^ solution of azide-coumarin and for MCC-P_NH_ after azide-coumarin attachment.
