# Supplementary material for: Optimization of the Heterogeneous Synthesis Conditions for Cellulose Tosylation and Synthesis of a Propargylamine Cellulosic Derivative
Source: Polymers (Basel). 2024 Dec 29;17(1):58. doi: 10.3390/polym17010058 (PMC11722636; doi:10.3390/polym17010058)
Supplement: Supplementary file 1 [file polymers-17-00058-s001.zip › polymers-3371985-supplementary.pdf]

Optimization of the heterogeneous synthesis conditions for cellulose tosylation and synthesis of a propargylamine cellulosic derivative

Marcos V. Ferreira [1]*, Poliana Ricci [1], Henrique A. Sobreira [1], Anizio M. Faria [1,2], Rodrigo B. Panatieri [2], Brent S. Sumerlin [3], Rosana M. N. Assunção [1,2]

[1] Institute of Chemistry, Federal University of Uberlândia, Uberlândia 38400-902, Brazil
[2] Institute of Exact and Natural Sciences, Federal University of Uberlândia, Ituiutaba 38304-402, Brazil
[3] George & Josephine Butler Polymer Research Laboratory, Department of Chemistry, University of Florida, Gainesville 32608, United States of America

* To whom correspondence should be addressed: ferreira.marcosvinicius88@gmail.com

SUPPLEMENTARY MATERIALS

ELEMENTAL ANALYSIS

Elemental analysis (EA) was applied to some samples produced during the experimental design and optimization tests to determine sulfur percentage (S%). The EA data established a linear correlation between the S% and the AR, calculated from the FTIR spectra. This correlation was observed in an AR range of 0.53 to 0.89, with an R^2 and an adjusted R^2 of 0.96438 and 0.95844, respectively (Fig. S1). Equation 5 expresses the DS_{tos} linear correlation.

$$DS_{tos} = (AR - 0.14633)/0.48356 \qquad (S1)$$

The DS was chosen as the response factor in the Doehlert matrix. To estimate it, we adapted a method devised by Rahn *et al.* (1996)[26] based on the correlation of the absorbance ratio (AR) for absorptions at 1174 cm^{-1} (attributed to the SO_2 bond from the tosyl group) and at 1056 cm^{-1} (corresponding to C-O-C stretching in the cellulose backbone). These measurements were

obtained by FTIR-ATR spectroscopy and were correlated with the sulfur percentage determined by elemental analysis (see Table S1 and Fig. S1 of Supporting Information). This approach allowed us to use a fast, reliable, and cost-effective technique, such as FTIR-ATR spectroscopy, to estimate the DS for the tosylate samples (DS_{tos}).

Therefore, the calculated DS for the sample produced under extrapolated optimized conditions was 1.80, and this sample was used as an intermediate in the aminopropargylation reactions of cellulose.

Table S1. Estimated degree of substitution by elemental analysis of some selected tosyl cellulose samples and their respective absorbance ratio (AR) for the absorptions at 1174/1056 cm^{-1}.

Sample	Weight (mg)	%S	nS (µmol)	1174/1056 cm^{-1}	DS
Exp 13	2.717	2.871	2.433	0.4416	0.5593
MCC-Tos 4.5:1-48h	2.259	3.802	2.678	0.4969	0.7407
Exp 12	2.584	3.845	3.098	0.5294	0.7491
MCC-Tos 5:1-72h	2.848	4.693	4.169	0.5629	0.9144
MCC-Tos 7:1 74 h	2.443	5.630	4.297	0.6472	1.099
MCC-Tos 7:1-96h	2.442	6.714	5.113	0.7486	1.308
MCC-Tos 7:1-144 h	2.771	7.336	6.340	0.8774	1.429

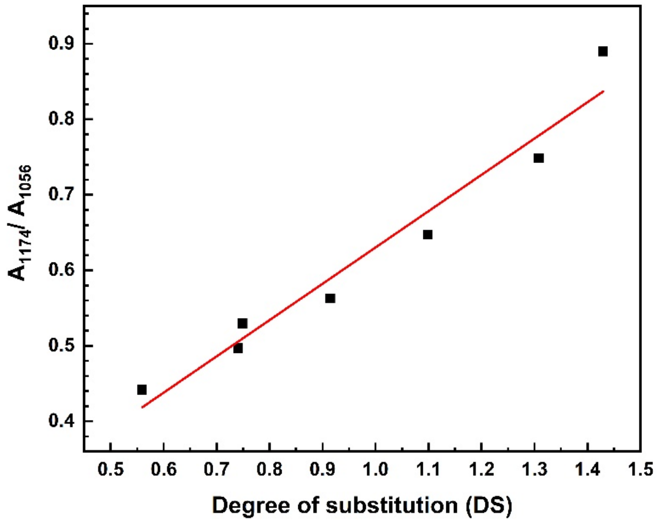

Figure S1. Absorbance ratio for absorptions at 1174/1056 cm^{-1} as a function of the estimated degree of substitution obtained by elemental analysis of the sulfur content (correlation coefficient 0.9784).

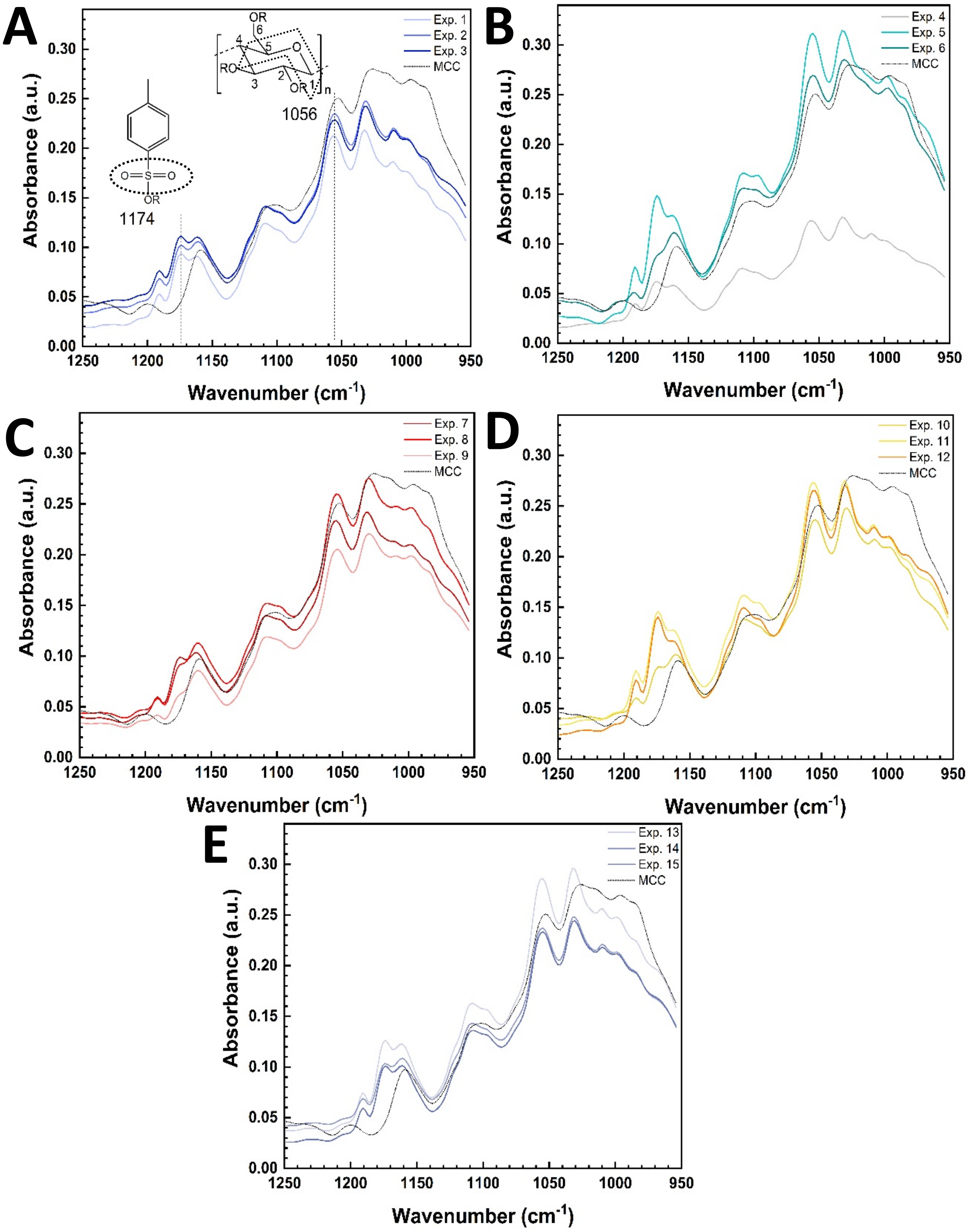

Figure S2. FTIR spectra in the 1250 to 950 cm^{-1} range for the tosyl celluloses synthesized under the different experimental conditions established by the Doehlert Matrix. R represents cellulose at 1174 cm^{-1} and Tos or H at 1056 cm^{-1}.

Table S2. Estimated coefficients of the quadratic model were obtained according to the Doehlert matrix for cellulose tosylation optimization.

Coefficients	Value	Error	t-test	*p*-value
Constant	0.437	± 0.016	28.141	0.0111*
x_1	-0.031	± 0.013	2.303	0.0695
x_2	0.088	± 0.013	6.536	0.0012*
x_3	0.075	± 0.013	5.578	0.0025*
x_1x_1	0.018	± 0.024	0.725	0.5008
x_2x_2	-0.021	± 0.024	0.851	0.4334
x_3x_3	-0.008	± 0.023	0.359	0.7344
x_1x_2	-0.042	± 0.031	1.364	0.2308
x_1x_3	0.074	± 0.035	2.146	0.0846
x_2x_3	0.024	± 0.035	0.708	0.5103

* Significant at the 95% confidence level ($p < 0.05$).

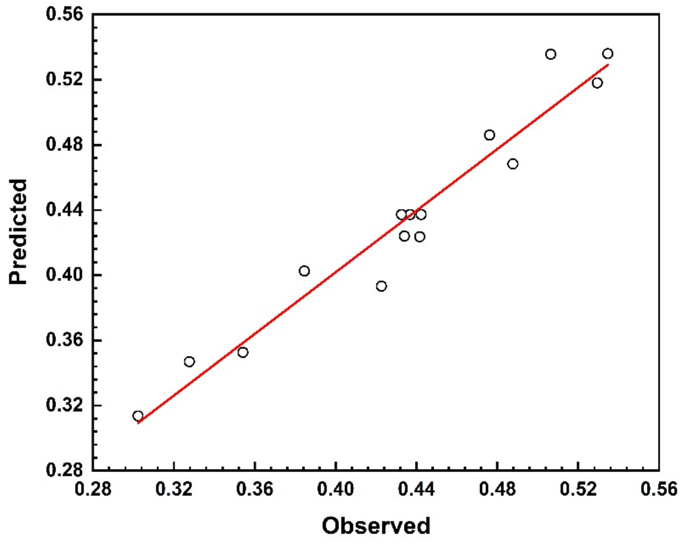

Figure S3. A plot of the absorbance ratio at 1174/1056 cm-1 as predicted by the Doehlert Matrix versus the values observed in the FTIR spectra (linear correlation of 0.9417).

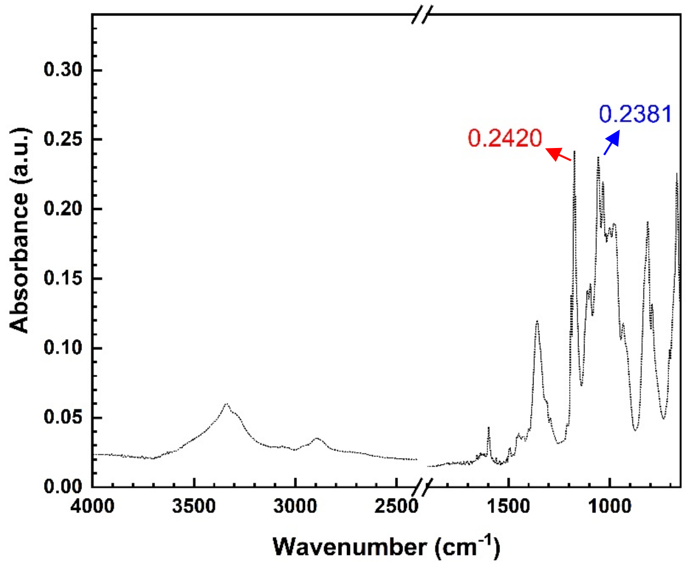

Figure S4. FTIR spectrum for the tosyl cellulose synthesized at room temperature with a reaction time of 144 h and a molar ratio of 10:1 (TosCl:AGU).

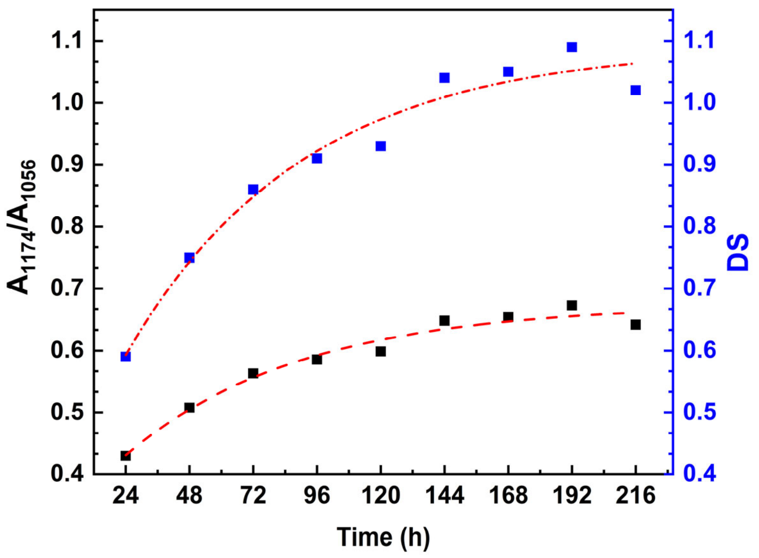

Figure S5. The behavior of the absorbance ratio at 1174/1056 cm^{-1} and the estimated DS values over time for MCC tosylation at a 5:1 molar ratio (TosCl:AGU).

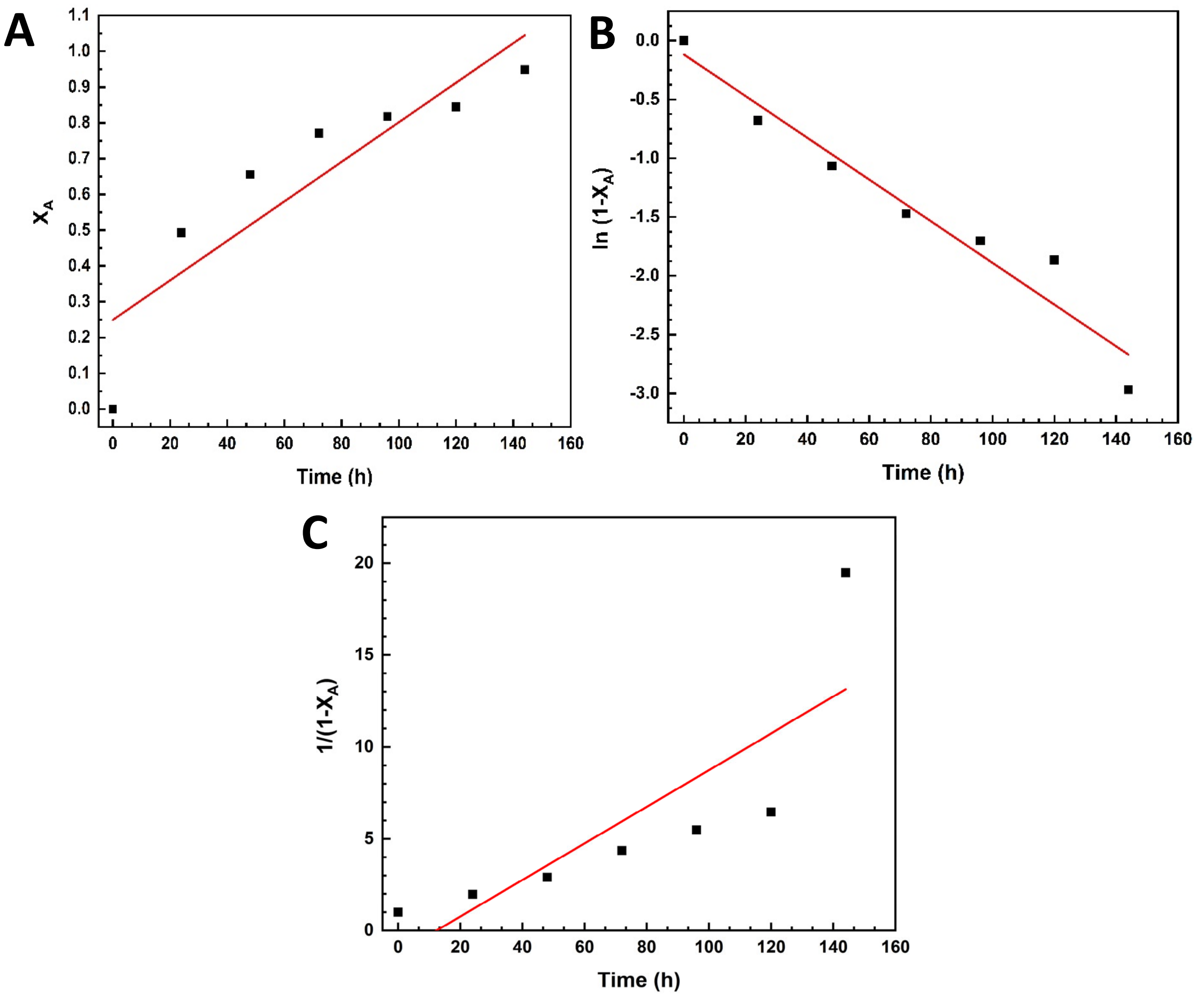

Figure S6. The application of three pseudo-order models: A) zero, B) first, and C) second-order for the tosylation of MCC after reaching equilibrium at 144 h.

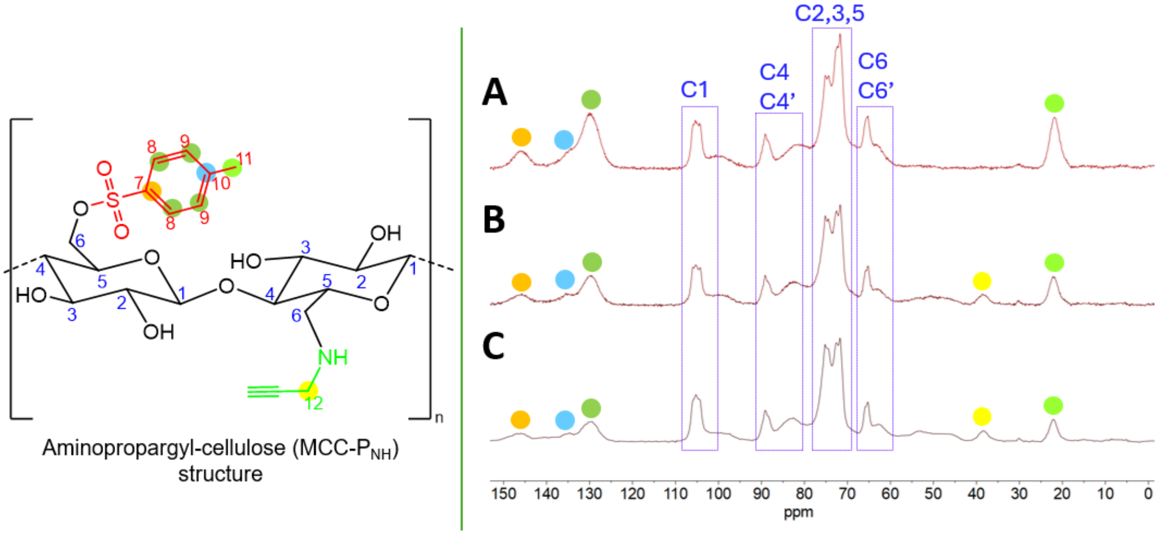

Figure S7. CP/MAS ^{13}C-NMR spectra of amino propargyl-celluloses (MCC-P$_{NH}$): A) after 24 h; B) 48; and C) 168 h of amino propargylation reaction.

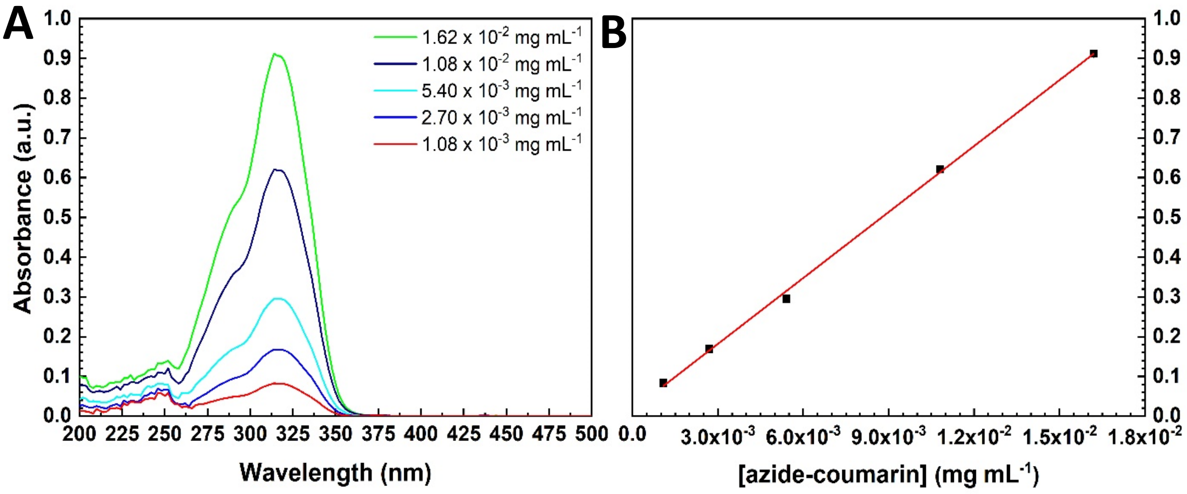

Figure S8. A) UV-vis spectra for different concentrations of azide-coumarin; B) standard curve for the azide-coumarin in mg mL^{-1}, with r and R^2 equal to 0.99949 and 0.99863, respectively.

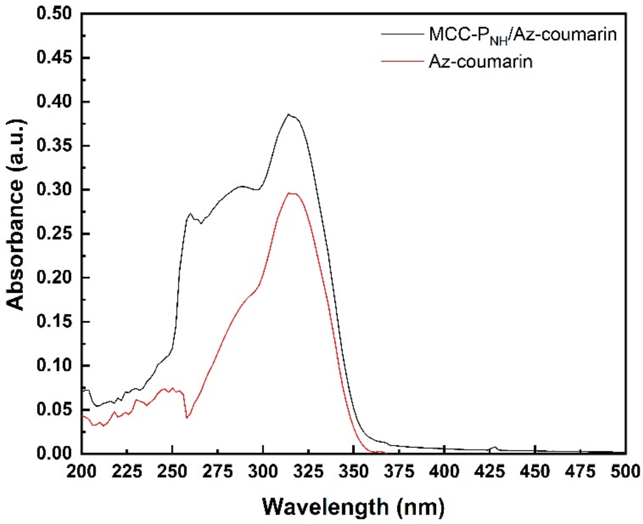

Figure S9. Comparative UV-vis spectra for a 5.4×10^{-3} mg mL^{-1} solution of azide-coumarin and for MCC-P_{NH} after azide-coumarin attachment.
